# Laparoscopic paediatric inguinal hernia repair: lessons learned from 102 cases

**DOI:** 10.1007/s11845-022-02975-2

**Published:** 2022-03-22

**Authors:** Lukas O’Brien, Enda Hannan, Sinead Hassett

**Affiliations:** 1 Department of Paediatric Surgery, Children’s Health Ireland at Crumlin, Dublin, Ireland; 2 Royal College of Surgeons in Ireland, Dublin, Ireland

**Keywords:** Inguinal hernia, Laparoscopic hernia repair, Paediatric hernia

## Abstract

**Introduction:**

Paediatric inguinal hernias (IHs) are common. The first paediatric laparoscopic hernia repair was described by El-Gohary and colleagues in the United Arab Emirates in 1993. Both laparoscopic inguinal hernia repair (LIHR) and open repair still exist concurrently with no consensus on gold standard treatment at present. The purpose of this study was to retrospectively evaluate our initial experience with LIHR in paediatric patients.

**Methods:**

A retrospective observational cohort study of all paediatric patients that underwent LIHR in our institution was performed. Intraoperative and postoperative outcomes were examined.

**Results:**

During the study period, 102 patients were scheduled for LIHR. The majority (76.5%) were male with a median age of 5 months. Thirty two patients (31.4%) were neonates at the time of surgery. The majority of cases (83.3%) were elective procedures. There were no instances of intraoperative vascular or visceral injury. Most patients underwent surgery as a day case. Eighteen patients underwent bilateral LIHR. The recurrence rate was 1.9%. These occurred in the first two patients to undergo LIHR, after which no recurrences were observed following a modification of the technique. The overall complication rate was 7.1%, most of which were managed conservatively.

**Conclusion:**

Paediatric LIHR is a safe, feasible and effective procedure that is associated with a short inpatient length of stay, a low recurrence rate and low postoperative complication rate. The technique is versatile and can be used to treat both elective and emergency presentations with IH in a wide age range.

## Introduction

Inguinal hernias (IHs) are common in the paediatric population with a reported incidence ranging from 3 to 5% in term infants and up to 13% in infants born before 33 weeks of gestation [1, 2]. The majority of these are indirect, arising due to failure of the processus vaginalis to close [3]. Due to the risk of incarceration and gonadal infarction, timely surgical repair is essential [4]. The aim of surgery is to close the patent processus vaginalis (PPV) at the level of the deep ring without damaging the spermatic cord structures contained within the inguinal canal [5]. Traditionally, this has been performed by an open approach, utilising a groin incision through which the hernia sac is dissected free from the spermatic cord and suture ligated at the deep ring [5]. However, open inguinal herniotomy is not without disadvantages. These include wound haematoma or seroma, hernia recurrence and damage to the vas deferens or the testicular blood supply [6, 7].

The advent of laparoscopic surgery has offered many advantages over conventional open surgery [8] Laparoscopic inguinal hernia repair (LIHR) may identify a subclinical contralateral PPV, which will allow for simultaneous repair, preventing later contralateral IH development and thus obviating the need for subsequent surgery [9]. This is particularly advantageous in the premature population where general anaesthesia carries a higher risk of post-operative apnoea and need for post-operative ventilation as a result of reduced lung compliance secondary to bronchopulmonary dysplasia typical in this patient cohort.

It is also a valuable option for recurrent IH in children following previous open inguinal hernia repair (OIHR), where further open surgery is challenging with a significant risk of damaging the vas deferens or testicular vessels [10]. Despite these advantages, LIHR in children remains controversial and uptake has been slow, with some evidence suggesting an association with a longer operative time and higher recurrence rate [11, 12].

The purpose of this study was to retrospectively evaluate intraoperative and postoperative outcomes in paediatric patients that underwent IH repair by a standardised laparoscopic technique in a high-volume specialist paediatric centre. From this, we aimed to determine if LIHR in paediatric patients represents a safe, feasible and effective alternative to OIHR that will allow this patient population to benefit from the advantages of minimally invasive surgery.

## Methods

### Study design and data collection

A retrospective observational cohort study was performed of all paediatric patients, defined as 16 years of age or under, who presented to the service with IH and subsequently underwent LIHR. This was a single-centre study with all procedures performed by a consultant paediatric surgeon on the specialist division of the medical register or by a senior paediatric surgery trainee under scrubbed consultant supervision. During this study period, all patients who presented to this consultant’s service with IH were managed by this technique, with all being included in this study. Patients who underwent LIHR were identified from operating theatre logbooks and data was collected by reviewing patient medical records, operative notes and outpatient clinic letters. Demographic, intraoperative and postoperative data was collected.

### Surgical technique and postoperative care (Figs. 1, 2, 3, 4)

In all cases, the procedure was performed with the patient in supine position under general anaesthetic. In children under the age of 2 years, 3-mm laparoscopic instruments were used, with 5-mm laparoscopic equipment used otherwise. An intraperitoneal approach was used with umbilical access gained using a curvilinear subumbilical incision followed by a standard Hasson technique for port insertion and establishment of pneumoperitoneum. Two further small incisions were placed in the left and right flank under direct vision through which the surgeon’s instruments were placed directly. The patient position was changed to 45° Trendelenburg position to aid in reduction of hernia contents and visualisation of both internal rings. Both deep rings were instrumented to confirm the diagnosis of IH and to assess for the presence of contralateral PPV or IH. Hernia contents were reduced from the inguinal canal into the peritoneal cavity using gentle traction by atraumatic graspers, and the contents were then inspected to ensure viability. Monopolar diathermy of the peritoneum at the superior aspect of the internal ring was performed. Following this, the peritoneum around the open internal ring was closed by purse-string technique using a 4.0 nonabsorbable monofilament suture (Surgipro^™^ Monofilament Polypropylene, Covidien^™^, Medtronic Inc.). The knot was tied in an intracorporeal manner. As the knot was being tied, the assistant applied pressure to the groin and scrotum to ensure that complete scrotal extravasation of CO2 was achieved. Following this closure, the area was inspected to ensure no further extravasation of CO2 had subsequently occurred, which would indicate that the purse-string ligation was not optimal. The contralateral open deep ring was closed in the same manner if present in the under 6-month population.

The fascia was closed following removal of the umbilical port by 4.0 Polysorb^™^ suture and skin glue (Histoacyl glue, Indermil Flexifuse^™^, Connecticon Medical Inc.) was used to close bilateral lumbar stab incisions and the umbilical skin incision. Patients were discharged on the day of surgery if well, except in the case of neonates under 60 weeks corrected gestational age, who stayed for postoperative monitoring overnight as per anaesthesiology departmental guidelines. All patients were reviewed in the outpatient department 6 weeks postoperatively.

### Statistical analysis and research ethics

All patient data was anonymised for the purpose of this study. No identifying information was retained by the authors or included in this article. Data was analysed using basic descriptive statistics. As this was a retrospective service evaluation involving anonymised data, ethics committee approval was not required in our institution.

## Results

### Patient demographics (Table 1)

**Table 1:** Patient demographics

**Patient demographics**	
*Median age*	5 months (range 2 days to 12 years)
*Male (%)*	76.5% (*n* = 78)
*Female (%)*	23.5% (*n* = 24)
*ASA I (%)*	68.6% (*n* = 70)
*ASA II (%)*	31.4% (*n* = 32)
*ASA III-V (%)*	0% (*n* = 0)
*Unilateral groin symptoms (%)*	90.2% (*n* = 93)
*Bilateral groin symptoms (%)*	8.8% (*n* = 9)
*Elective presentation (%)*	83.3% (*n* = 85)
*Emergency presentation (%)*	16.7% (*n* = 17)

Between January 2018 and November 2020, 102 paediatric patients were scheduled for LIHR. The majority of patients were male (76.5%, *n* = 78) and the median age was 5 months (range 2 days to 12 years). All patients had an American Society of Anaesthesiologists (ASA) grade of either I (68.6%, *n* = 70) or II (31.4%, *n* = 32). Most cases were elective procedures (83.3%, *n* = 85) for symptomatic IH, while the remaining cases were emergency presentations with suspected incarceration (16.7%, *n* = 17). All patients had a pre-operative clinical diagnosis of IH made without the use of ultrasonography prior to surgery. The majority (90.2%, *n* = 93) were referred with unilateral IH on presentation, with the remainder (8.8%, *n* = 9) having clinical features of bilateral IH. Thirty two patients (31.4%) were neonates at the time of surgery.

### Intraoperative outcomes (Table 2)

**Table 2:** Intraoperative and postoperative outcomes

**Intraoperative and postoperative outcomes**	
*Complication rate (%)*	7.1% (*n* = 7)
*Clavien-Dindo I (%)*	4.1% (*n* = 4)
*Clavien Dindo III (%)*	3% (*n* = 3)
*Clavien-Dindo II, IV, V (%)*	0% (*n* = 0)
*Conversion to open surgery (%)*	(*n* = 2)
*Minimal estimated blood loss (%)*	100% (*n* = 99)
*Visceral/vascular injury (%)*	0% (*n* = 0)
*Port site seroma (%)*	2% (*n* = 2)
*Port site hernia requiring surgery (%)*	1% (*n* = 1)
*Recurrent IH (%)*	2% (*n* = 2)
*Chronic pain (%)*	0% (*n* = 0)
*Testicular atrophy (%)*	0% (*n* = 0)
*30-day readmission (%)*	0% (*n* = 0)
*30-day reoperation (%)*	0% (*n* = 0)
*30-day mortality (%)*	0% (*n* = 0)

Unilateral indirect IH was noted in 79% (*n* = 81) of cases and bilateral indirect IH in 17.6% (*n* = 18), all of which went on to have laparoscopic repair. Of the remaining three procedures, one case yielded no laparoscopic evidence of IH and was thus concluded as a diagnostic laparoscopy, while the other two patients were diagnosed with direct IH and were converted to open groin exploration and a standard Bassini repair of the posterior wall of the inguinal canal. Thus, the rate of conversion to open surgery was 1.9% (*n* = 2), with 99 patients undergoing LIHR. There were no instances of intraoperative vascular or visceral injury. Iatrogenic injury to spermatic cord structures was not observed in any case.

### Postoperative outcomes (Table 2)

The majority of patients who underwent LIHR were discharged from the hospital on the day of surgery (57.6%, *n* = 57). The remaining patients (42.4%, *n* = 42) stayed in hospital for one night. Of these, the majority (76.2%, *n* = 32) were neonates that were scheduled pre-operatively to stay overnight for the purposes of apnoea monitoring. The median length of stay was 0 nights (range 0–1 nights). Two patients (2%) who underwent LIHR went on to subsequently develop a recurrent IH. These patients were the first two cases of LIHR performed in this cohort. Two patients (2%) developed umbilical port site haematomas postoperatively that resolved with conservative management. One patient (1%) developed an umbilical port site hernia that required surgical repair due to significant pain around the umbilicus. Two further patients initially suspected to have developed umbilical port site hernias resolved without any surgical intervention. At 6-week follow-up, no patients had evidence of testicular atrophy or raised concerns related to a reduced testicular volume. There were no cases of ascending testes postoperatively. The overall complication rate was 7.1% (*n* = 7). No 30-day readmissions, reoperations or mortalities occurred.

## Discussion

In the current study, we retrospectively reviewed the outcomes of our initial experience with LIHR for paediatric patients in a high-volume tertiary referral centre. It was observed that LIHR is a safe, feasible and effective means of treating both elective and emergency presentations of IH in the paediatric population. The postoperative complication rate was low. No adverse intraoperative events occurred with a prompt postoperative recovery occurring in all patients. The technique was shown to be transferrable and reproducible, with paediatric surgical trainees gaining the ability to perform the technique under scrubbed consultant supervision.

Only two patients developed recurrent IH during the study period. It is important to note that these two recurrences occurred in the first two patients that underwent LIHR in our institution. Initially, the technique utilised did not involve diathermy of the peritoneum at the superior aspect of the internal ring to encourage reperitonealisation [13]. However, after these two early recurrences were noted, the technique was altered to include this step. Following the implementation of this technical modification, no recurrences have been observed since.

LIHR offers a number of distinct advantages compared to OIHR [6, 8]. As groin incision and exploration is avoided in the laparoscopic technique, groin-related complications such as scrotal haematoma and wound seroma have been demonstrated by systematic review to be less frequently observed in LIHR compared to open surgery [14]. LIHR is also advantageous in cases of bilateral IH, allowing both sides to be repaired without the need for extra incisions unlike in open surgery. This results in a shorter operative time improved cosmetic outcomes and reduced postoperative pain [15]. Laparoscopy also serves as a diagnostic procedure and may allow for the detection and repair of an incidentally detected IH or PPV on the contralateral side in patients with unilateral symptoms, thus avoiding the need for future surgery [16]. Laparoscopic herniotomy can also reliably identify the presence of direct inguinal hernia or less commonly a femoral hernia. In our series, 2 patients had a direct inguinal hernia and no femoral hernias were identified.

It is well recognised that re-operative surgery for a recurrent unilateral IH can be highly challenging and hazardous due to scar formation within the inguinal canal [17]. In such scenarios, a surgical approach that avoids the previous route is desirable. Therefore, recurrence after OIHR is better repaired by LIHR and vice versa [18]. In patients with large hernia sacs (defined as a neck greater than 3 cm in diameter), it has been recommended that laparoscopic repair is superior, as groin dissection in such cases poses an increased risk of scrotal haematoma and injury to spermatic cord structures [19]. This is particularly relevant in the premature neonatal population where the thin-walled sac can create a technical challenge for even the most experienced surgeon. LIHR is also advantageous in emergency presentations, allowing for reduction of strangulated intestine by widening the internal ring and facilitating careful intraperitoneal inspection post-reduction to assess for visceral viability [19, 20]. It also allows for earlier repair, as traditionally a period of 24-h post reduction has been recommended to allow the oedema of the hernia sac to reduce prior to OIHR. In our series, 16.7% of cases were performed following emergency presentations. In one small series, serious complications were observed in 8% of emergency OIHR compared with 1.6% in emergency LIHR [20]. Furthermore, it has been observed that LIHR is advantageous in the presence of obesity, ambiguous genitalia and undescended testes [19, 21].

Despite these advantages, the uptake of LIHR has been relatively slow, with a recent international survey demonstrating that 83% of paediatric surgeons still prefer to perform OIHR [22]. Paediatric LIHR has faced numerous criticisms which may explain the resistance to its utilisation [19]. A recent systemic review based on four randomised controlled trials and one prospective study demonstrated no statistically significant difference in postoperative pain scores, inpatient length of stay or recurrence rates, concluding that there is no evidence that clinical outcomes improve with LIHR in paediatric patients [23]. The cost of treatment for LIHR may also be up to three times as expensive as OIHR depending on instrumentation utilised [24]. Unlike OIHR, LIHR invariably requires a general anaesthesia and the creation of pneumoperitoneum which may impede venous return and thus pose elevated risk, especially in preterm infants and in the presence of cardiorespiratory disorders [19]. Finally, the perception of a steep learning curve has been a deterrent for performing LIHR [19]. However, a recent study suggested that a consultant paediatric surgeon with laparoscopic experience could gain competency in the technique after 13 cases [25].

It is clear that there still exists contentious debate in the literature regarding the utilisation of laparoscopy for the repair of paediatric IH without a definitive consensus [19, 23, 26]. A recent literature review concluded that, in the absence of clear superiority of one approach over the other, the choice of repair may be determined by surgeon preference [19]. However, whichever technique is utilised, it should result in a short length of stay, a low recurrence rate and low postoperative complication rate [19]. Our results demonstrate that our technique meets these criteria.

Our study is not without limitations. The study is retrospective in nature with all cases performed by a single surgeon. While we have demonstrated favourable postoperative outcomes following LIHR, we are unable to evaluate how these results compare to OIHR due to the lack of a control group. Our study is also limited by a short follow-up period and lack of long-term data, creating the potential for missed late complications, such as subsequent hernia recurrence, testicular atrophy or testicular ascent. Despite these limitations, we have demonstrated a high rate of success in the utilisation of LIHR in paediatric patients, with no recurrences observed after an early modification to the technique. Based on this information, LIHR may represent a safe, feasible and effective means of treating paediatric IH. Given the current lack of consensus in the literature regarding such an approach, further information reporting on laparoscopic techniques in paediatric patients is of value. This study also provides valuable knowledge for those embarking on initial experience with paediatric LIHR. 

## Conclusion

Our initial experiences with paediatric LIHR demonstrate that it is a safe, feasible and effective procedure that is associated with a low recurrence rate and a low postoperative complication rate. The technique is versatile, having been successfully used to treat both elective and emergency presentations with IH in a wide age range.

**Fig. 1:**
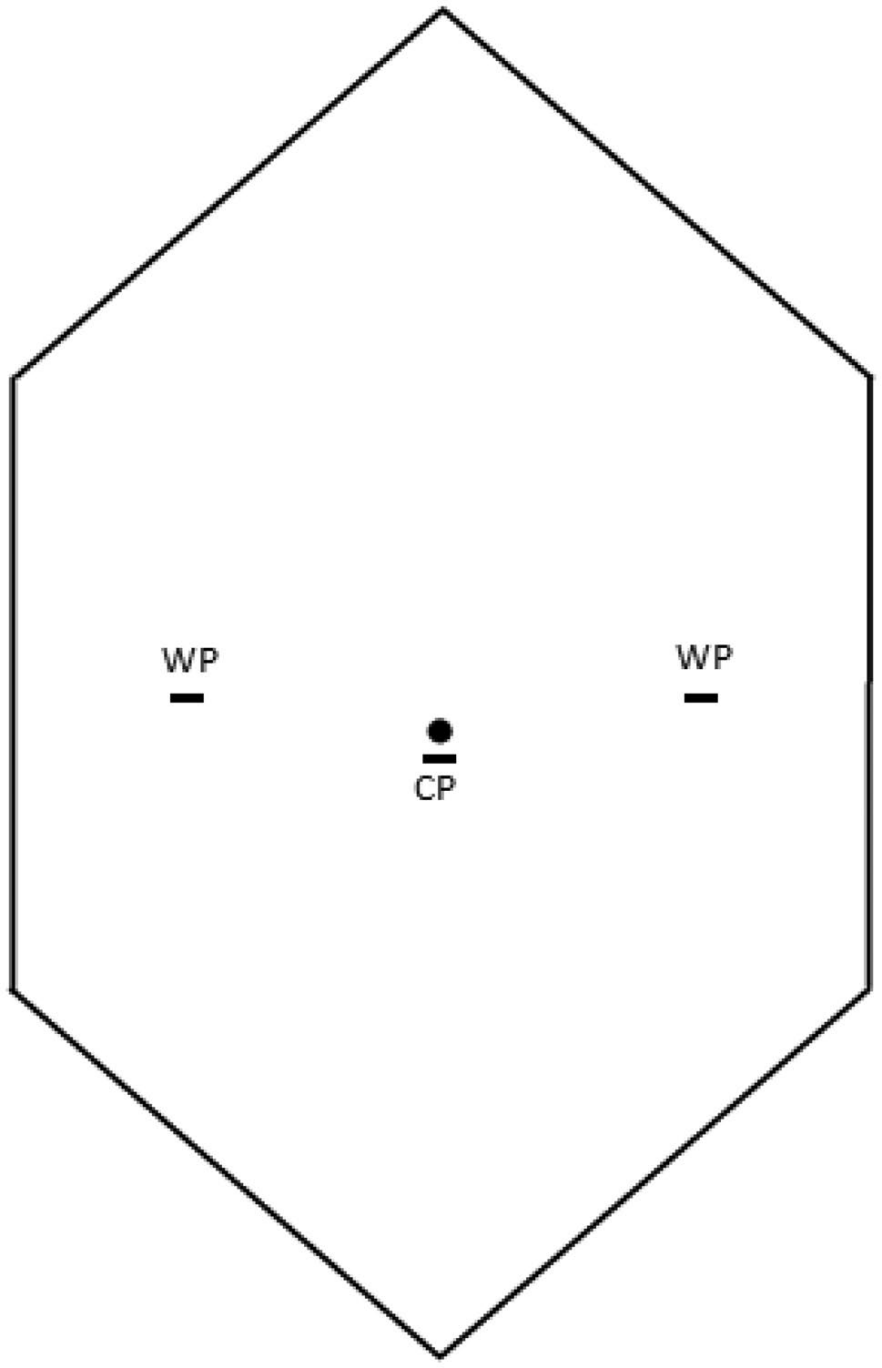
Port placement utilised for paediatric LIHR. Three millimeters of ports was used in children under 2 years, and 5-mm ports in those 2 years and older. CP, camera port, WP, working port

**Fig. 2:**
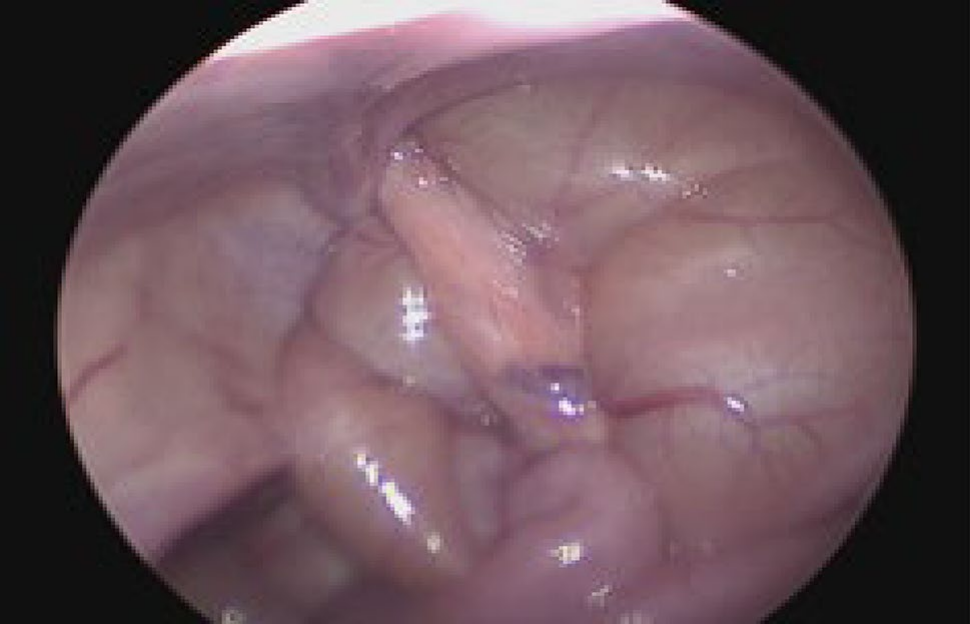
Small bowel-containing indirect inguinal hernia

**Fig. 3:**
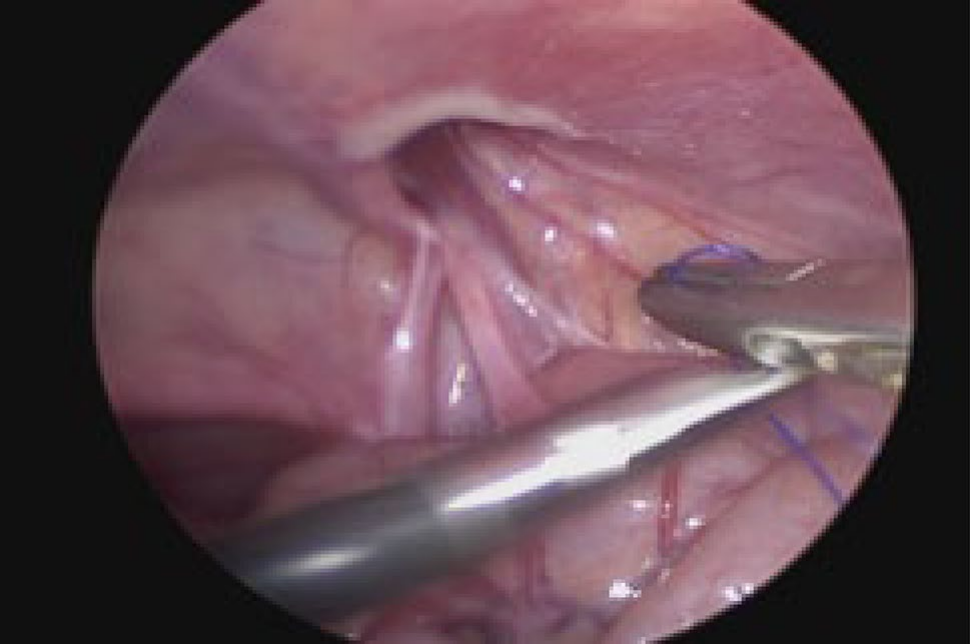
Indirect inguinal hernia defect at the internal ring following reduction of hernia contents

**Fig. 4:**
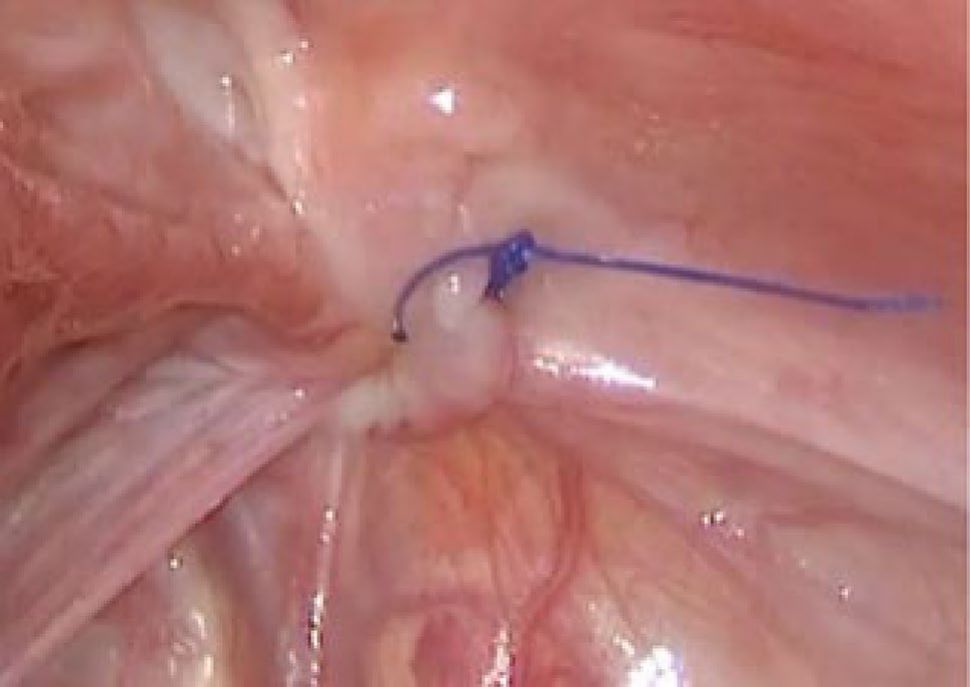
Completion of the purse-string suture at the neck of the hernia sac

## Data Availability

The data that supports the findings of this study is available from the corresponding author upon reasonable.

## Abbreviations

IH: Inguinal hernia
PPV: Patent processus vaginalis
LIHR: Laparoscopic inguinal hernia repair
OIHR: Open inguinal hernia repair
